# Effect of the board game as educational technology on schoolchildren’s knowledge on breastfeeding^1^

**DOI:** 10.1590/1518-8345.2316.3049

**Published:** 2018-09-03

**Authors:** Fernanda Demutti Pimpão Martins, Luciana Pedrosa Leal, Francisca Márcia Pereira Linhares, Alessandro Henrique da Silva Santos, Gerlaine de Oliveira Leite, Cleide Maria Pontes

**Affiliations:** 2 Doctoral student, Universidade Federal de Pernambuco, Recife, PE, Brazil. Scholarship holder at Coordenação de Aperfeiçoamento de Pessoal de Nível Superior (CAPES), Brazil.; Coordenação de Aperfeiçoamento de Pessoal de Nível Superior, Brazil; 3 PhD, Adjunct Professor, Departamento de Enfermagem, Universidade Federal de Pernambuco, Recife, PE, Brazil.; 4 MSc, Assistant Professor, Departamento de Enfermagem, Universidade Federal de Pernambuco, Recife, PE, Brazil.; 5 Master’s student, Universidade Federal de Pernambuco, Recife, PE, Brazil.; 6 PhD, Full Professor, Departamento de Enfermagem, Universidade Federal de Pernambuco, Recife, PE, Brazil.

**Keywords:** Breast Feeding, Child, School Health, Clinical Trial, Educational Technology, Nursing

## Abstract

**Objective:**

to evaluate the effect of the board game as an educational technology on schoolchildren’s knowledge on breastfeeding.

**Method:**

cluster-randomized clinical trial, held in nine schools, with 99 children in the third grade of elementary school (control group = 51 and intervention group = 48). The pretest was conducted in both groups; intervention consisted in the application of the educational technology immediately after pretest to the intervention group; and the post-test was applied on the 7th and 30th days to both groups. For the analysis of children’s knowledge on breastfeeding, we considered the pre- and post-test score means, using the Mann-Whitney test - for comparing the means between groups - and the Wilcoxon test - within the same group.

**Results:**

there was no statistically significant difference between the groups in the pretest. In the follow-up, when comparing the groups, there were higher means in the intervention group, on the 7th (19.68 ±1.788) and on the 30th (20.16±1.260) days, with statistically significant difference. Within the intervention group, there was significant increase of the means in the pretest (15.89±3.082) for the 30th day (20.16±1.260).

**Conclusion:**

such educational intervention has significantly contributed to the increase in scores of children’s knowledge on breastfeeding for the intervention group. UTN: U1111-1184-7386.

## Introduction

Worldwide, despite the benefits of breastfeeding for children’s and women’s health, considering its economic and environmental advantages, only 37% of children younger than six months are exclusively fed with breast milk. Several factors can affect this practice, among which outstands the influence of the family - the primary network. Therefore, actions for promoting breastfeeding should include the family and be invested since early ages, during childhood, enabling the promotion of the positive culture of breastfeeding^1^ ^-^ ^5^.

In the primary network, exposing the child to the practice of breastfeeding, at home or in public environments, can be an opportunity to learn, whereas the school - the secondary network - is a formal learning space able to complement children’s knowledge acquired within family and social contexts^6^. This may favor the awareness of this practice as appropriate, encourage young people to choose healthier behaviors, and contribute to the success of breastfeeding^6^ ^-^ ^7^.

The implementation of educational interventions in school demonstrates a positive effect on students’ knowledge concerning breastfeeding. There are several pedagogical strategies: classes with active participation, reading activities, videos, Q&A games, and role-playing stories^7^ ^-^ ^9^. However, we note that, among these technologies, the board game was not employed.

This type of game has been applied to the themes of oral health, prevention of diseases (dengue fever), and quality of life with positive results in the increased knowledge of children^10^ ^-^ ^12^. This indicates that it can be an effective tool in teaching contents comprising breastfeeding for schoolchildren.

Constructivist theories support the educational potential of games for the children’s cognitive development, stimulated by the competitive spirit and the interaction with adults and peers more capable than them^13^ ^-^ ^14^. In this context, board games emerge as an active and playful pedagogical strategy, able to motivate students in learning about breastfeeding. Therefore, we aim to evaluate the effect of the board game as educational technology on schoolchildren’s knowledge on breastfeeding. 

## Method

Cluster-randomized clinical trial, two treatment groups, single blind, held in the period from June 20 to December 16, 2016, in nine Municipal Public Schools of the IV Health District (from Portuguese, *Distrito Sanitário* - DS IV) in Recife, Pernambuco, Brazil. 

The population was composed of children enrolled in the third grade of elementary school of such district. The choice was based on the third period of cognitive development (concrete operations), which corresponds to children aging between 7 and 10 years. At this stage, there is loss of egocenter, greater tendency for socialization, development of the capacity to perform logical relations of thought, greater understanding and respect for the rules, and evolution of games as collective activities^13^ ^-^ ^14^. These features meet the educational technology applied in our study.

The sample consisted of third graders studying at municipal public schools of the city of Recife, Pernambuco, with regular attendance in the collection period, aging between 7 and 10 years, able to read words and sentences, indicated by the teacher of the class. Children on medical leave due to illness or with disabilities, identified by the teacher, were excluded from the research. Student dropout, or leave of absence due to medical reasons, and children who did not participate in all steps of the research were considered losses.

The pilot study was carried out on 20 children from the third grade of elementary school, being 10 in each group (control - CG and intervention - IG), in two municipal schools of the DS IV, drawn at random. The purpose was to obtain data for the sample calculation, to clarify questions of the research team, and to verify the time required for conducting the interview and playing the game. These children composed the intervention study sample, because the pilot study followed the same steps of the clinical trial.

Research assistants, previously trained, were undergraduate and graduate students of the Nursing Programs of the Federal University of Pernambuco (UFPE). The training program, with course load of 10 hours, was offered by the lead researcher in two meetings with each assistant, individually or in small groups, in which the Standard Operating Procedure was handed out, which contained writing guidelines about the data collection. In these meetings, objectives, procedures, the instrument, and the research schedule were presented. In addition, research assistants performed the simulation of the instrument application, clarifying doubts and correcting errors in the conduct of the interview.

The team was divided into four subgroups, depending on the application of the test, namely: 1) CG pretest; 2) IG pretest; 3) CG post-test; and 4) IG post-test. Only one person was responsible for managing the intervention, which consisted in the application of the board game as an educational technology for children of the IG. This person also participated in the pretest collection in both groups.

The sample size was calculated based on the equation for two experimental means, in which the following were used: mean values (CG=18.2; IG=19.5) and standard deviation values (CG=2.97; IG=1.26) of scores of children’s knowledge on breastfeeding, verified in the pilot study, on the 7th day after pretest in both groups. Considering a confidence level of 95%, test power of 80%, the sample size comprised 96 children, estimating possible losses, added 20%, totaling 116 children (CG=58 and IG=58).

To minimize the risk of contamination, preventing CG and IG students from the same microregion, school, or class to be drawn, we opted for the cluster randomization. Randomization was performed in three steps with the aid of the Microsoft Office Excel^*®*^ program, using the reference of the Political-Administrative Microregion (from Portuguese, *Microrregião Político-Administrativa* - MPA) of DS IV and a numerical list of schools/classes of the third year of elementary school: 1) allocation of CG and IG according to the MPA: CG was allocated in MPA 4.1, and GI, in MPA 4.2 and 4.3; 2) simple random sampling for selection of schools in each group: in total, there were 9 schools, 5 for the CG and 4 for the IG; and 3) simple random sampling of the classes of the third year of elementary school: in the CG, 3 classes in the morning shift and 2 in the afternoon shift; and in the IG, 3 classes in the morning and 1 in the afternoon shift.

Data collection was performed using an instrument^15^ in a questionnaire format, created and validated for this research, structured in: 1) socioeconomic data (guardian/legal representative of the child) and breastfeeding-related data, which included independent variables; and 2) the children’s knowledge on breastfeeding, which contained 21 items (statements and illustrations), with response options “right,” “wrong,” and “I don’t know,” identified by adapted emotions^16^. For correct responses, one point was awarded, and for the wrong ones, or that which the student could not answer, zero. Thus, the total score could vary between 0 and 21 points. The outcome variable was the mean of the scores of children’s knowledge on breastfeeding, considering the support of the social network to breastfeeding women, verified in the CG and IG through the application of the post-test in the 7th and 30th days after the pretest. 

Initially, consent was obtained from the Municipal Department of Teaching of Recife, Pernambuco, Brazil, authorization from the school offices, and support of the teachers of the classes. In the individual meetings or in those with small groups, the authorization of the guardian/legal representative for children’s participation was required and socioeconomic data and previous history of children’s breastfeeding were collected.

Data collection proceeded in three steps:

*First step:* recruitment of scholars for assessing eligibility criteria. Children were individually invited to participate in the survey with the aid of a comic book. Then, we applied the instrument to assess children’s knowledge on breastfeeding in both groups (CG and IG) through an interview at some environment or private room. 

Children were guided as to the purpose of the study and that the answers to the instrument would not result in school scores or damages. The dates of the interviews were previously scheduled with the teacher to avoid harms to the school syllabus. The interview was held during school hours and had an average duration of 15 minutes. To minimize losses, all teachers have received a reminder with the dates of upcoming interviews, placed in the classroom at a visible place for the class.

*Second step:* the board game educational intervention *Trilha Família Amamenta* [Breastfeeding Family’s Trail], created and validated for our research, was held immediately after the pretest for the IG. In this group, according to the guidelines of the Principal of the school and the teachers, all the kids present in the classroom participated in the game. However, data were collected only from children whose guardians authorized such participation in the survey.

The first ten minutes were intended for the presentation of the game, goals, rules, and the distribution of the material among schoolchildren. Children were oriented to form groups of five and choose two leaders, responsible for the reading of question cards to their opponents. The homeroom teacher assisted in this step by distributing, between the groups, students with greater ease in reading texts. The game started and the research team aided the schoolchildren with the operation of the game, rules, clarification of doubts and, when necessary, in reading the question cards and texts of the board game. The game lasted about 50 minutes.

When the match finished, the material was collected and each children earned a game kit (1 board, 1 dice, 5 pins, 17 question cards, and the explanatory leaflet with rules), being oriented to take the material to their homes and play for a week with family members and friends. It was stressed that on the 7th day they would participate in another interview and, therefore, the date and the importance of their presence on the scheduled day were reinforced in order to proceed with the survey.

In both groups, teachers were guided not to discuss the content of breastfeeding at school to avoid bias in the study, and we considered that all children were naturally exposed to breastfeeding in their social network, through the contact with family, community, school, health services, and media. Therefore, scholars from the CG did not undergo intervention.

*Third step:* the post-test was applied to schoolchildren from the CG and the IG on the 7th and 30th day after the pretest. The time interval for the follow-up in similar studies varies in the literature, and there may be periods of one day, one month, three months, and up to six months^8^ ^,^ ^17^. In our study, the option for conducting the post-test on the 7th and 30th days after the pretest was based on literature and on the incentive to cognitive development mediated by this playful resource when enabling children to take the game to their homes and being encouraged to play with it during this period^13^ ^-^ ^14^.

Masking out of children in relation to allocation of groups was impossible due to the type of intervention - educational game. There was blinding of the research team responsible for collection in the CG, since their training was held at different times, separately from other research assistants, responsible for collection in the IG, in order to ensure the masking out of the allocation of groups for the assistants who participated in the collection of the CG. In the team assigned to collection in the IG, masking out was impossible, because the volunteers witnessed the application of the technology to the IG or were aware of the allocation of groups due to questions about children’s experience with the game. To minimize the risk of detection bias, the person who applied the educational intervention did not take part in the post-test collection. There was blinding of the person conducting the statistical analysis until the end of the assessment, identifying the groups by numbers - 1: control; and 2: intervention - in the database.

Data were typed in independent double entry, validated in the Epi Info^*®*^ program, version 3.5.2, and exported to the Statistical Package for Social Science (SPSS) software, version 20.0. 

The Kolmogorov-Smirnov test featured normal distribution of the groups regarding socioeconomic and breastfeeding-related characteristics. To test the homogeneity of the groups in relation to these variables and to the children’s experience with the game, we used the Chi-square test, for homogeneity, and the Fisher’s exact test, for comparing proportions of categorical variables. For continuous variables, we applied the Student’s t-test to those with normal distribution, and the Mann-Whitney test when noting anormality. 

In the assessment of children’s knowledge on breastfeeding, we found normality of the score means at baseline, using the Kolmogorov-Smirnov test. We used the Student’s t-test for comparison among groups. In the knowledge score on the 7th and 30th days, distribution of the score means was anormal, thus we applied the Mann-Whitney test for comparing knowledge between the groups and the Wilcoxon test for comparing the means in the same group between baseline and the 30th day. All findings were established considering the significance level of 5%.

For comparing the knowledge score of children and the variables sex, age, exposure to breastfeeding, and frequency with which the child played the game, we used the following tests: Student’s t-test, Mann-Whitney, Analysis of Variance (ANOVA), Kruskal-Wallis, and Wilcoxon.

The research was approved by the Research Ethics Committee of the Center of Health Sciences/UFPE, opinion no. 2,075,070, registered in the database of the *Registro Brasileiro de Ensaios Clínicos* [Brazilian Registry of Clinical Trials], under UTN number: U1111-1184-7386, and all the guidelines of the Consolidated Standards of Reporting Trials (CONSORT) were followed^18^.

## Results

**Figure 1:**
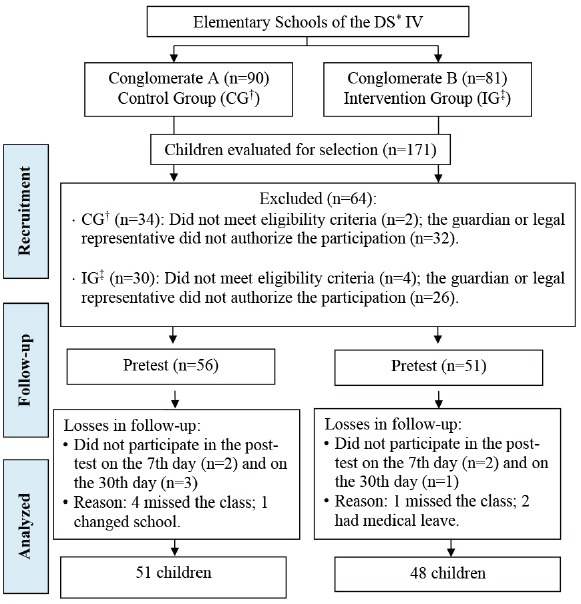
Flowchart of the steps of the experimental study on the schoolchildren’ knowledge on breastfeeding according to the CONSORT model^17^. Recife, PE, Brazil, 2016

In total, 171 children were assessed for eligibility, in the period from June 20 to November 1^st^, 2016, after cluster randomization of schools. The follow-up took place from September 1^st^ to December 16, 2016. At the end of the study, due to exclusion criteria and losses, 99 children participated (CG=51; IG=48), as depicted in Figure 1. 

*DS: Health District; †CG: control group; ^‡^IG: intervention group.

The groups were homogeneous regarding socioeconomic characteristics. The mean age of the children was 38.35 years (±10.34) for the CG and 39.42 years (± 11.82) for the IG. The mean of schooling years for the CG was 8.57 years (±3.75), and for the GI, 9.69 years (±4.18), as described in Table 1.

**Table 1:** Socioeconomic characterization of guardian/legal representative of the children according to research groups. Recife, PE, Brazil, 2016

Variables	Group		Total (n=99)	p-value
	Control (n=51)	Intervention (n=49)		
	n(%)	n(%)	n(%)	
Guardian/legal representative				
Kinship				
Mother	33(64.7)	31(64.6)	64(64.6)	0.681*
Father	9(17.6)	7(14.6)	16(16.2)	
Grandmother	5(9.8)	8(16.7)	13(13.1)	
Other	4(7.8)	2(4.2)	6(6.1)	
Marital status				
Single	17(33.3)	23(47.9)	40(40.4)	0.312*
Common-law marriage/married	29(56.9)	22(45.8)	51(51.5)	
Widow	2(3.9)	0(0)	2(2)	
Divorced	3(5.9)	3(6.3)	6(5.1)	
Schooling				
Did not attend school (illiterate)/knows how to read	4(7.8)	4(8.3)	8(8.1)	0.197*
Elementary School/Some Elementary School	30(58.8)	19(39.6)	49(49.5)	
High School/Some High School	16(31.4)	21(43.8)	37(37.4)	
College/Some College	1(2)	4(8.3)	5(5.1)	
Profession/occupation				
Housewife	21(41.2)	19(39.6)	40(40.4)	0.425^†^
Housekeeper	5(9.8)	9(18.8)	14(14.1)	
Other	25(49)	20(41.7)	45(45.5)	
Professional status				
Formal/Informal employment	17(33.3)	13(27.1)	30(30.3)	0.443*
Unemployed/receives benefits from the Government	31(60.8)	34(70.8)	65(65.7)	
Retired/gainful activity	3(5.9)	1(2.1)	4(4)	
Household income (BRL)^‡^				
<1MW^§^	17(33.3)	22(45.8)	39(39.4)	0.203^†^
≥1MW^§^	34(66.7)	26(54.2)	60(60.6)	
Number of people in the household				
<5	35(68.6)	36(75)	71(71.7)	0.482^†^
≥5	16(31.4)	12(25)	28(28.3)	
Number of children				
<3	37(72.5)	38(79.5)	75(75.8)	0.443^†^
≥3	14(27.5)	10(20.8)	24(24.2)	

*p-value of the Fisher’s exact test; †p-value of the Chi-square test for homogeneity; ^‡^family income whereas the minimum wage in the year 2016 was R$880.00 (BRL); ^§^MW: minimum wage.

Most children were male (CG=30 [58.8%]; IG=28 [58.3%]) and were born in Recife (CG=44 [86.3%]; IG=45 [93.8%]). The predominant age group was 8 years (IG=22 [45.8%]) and 9 years (CG=26 [51%]). According to the guardians, there was a preponderance of children who had been breastfed (CG=48 [94.1%]; IG=42 [87.5%]). Children themselves reported they were exposed to breastfeeding (CG=45 [88.2%]; IG=47 [97.9%]) and they knew they were breastfed (CG=44 [86.3%]; IG=41 [85.4%]). The groups demonstrated homogeneity regarding children’s socioeconomic and breastfeeding-related variables.

Concerning the scores of children’s knowledge on breastfeeding at baseline, there was no significant statistical difference between the CG and the IG. However, means verified in the follow-up, for the IG, showed higher values than those for the CG, with statistically significant difference between the groups in the 7th and 30th days. There was significant increase in the score means of children’s knowledge on breastfeeding between the baseline and the thirty-day time, both for the IG and the CG, according to Table 2. 

**Table 2:** Comparison between groups regarding mean values and standard deviation of the scores for knowledge on breastfeeding at baseline, on the 7th, and on the 30th days after intervention. Recife, PE, Brazil, 2016

Period	Groups				p-value
	Control		Intervention		
	Mean±SD*	CI^†^	Mean±SD*	CI^†^	
Baseline	16.08(±2.529)	15.37-16.79	15.89(±3.082)	15.00-16.79	0.747^‡^
7th day	17.59(±2.570)	16.87-18.31	19.68(±1.788)	19.17-20.21	0.000^§^
30th day	17.71(±2.773)	16.93-18.49	20.16(±1.260)	19.80−20.53	0.000^§^
p-value	0.000^║^		0.000^║^		

*SD: standard deviation; †CI: confidence interval; ^‡^Student’s t-test; ^§^Mann-Whitney U test; ^║^Wilcoxon test, considering the mean scores for knowledge verified at baseline and on the 30th day after intervention within the group.

In the results of the pre- and the post-test, for both CG and IG, evaluated by 21 items, we found that at baseline 2 items have shown statistically significant difference (3 and 7) and, on the 7th and 30th days, 8 items (3, 4, 6, 7, 10, 11, 12, and 13). In the pretest, we found hit percentage above 80% for the CG in 12 items, and for the IG, in 10 items. In the follow-up, we found on the 7th and on the 30th days 15 and 14 items for the CG, and 20 and 21 items for the IG, respectively, as evidenced in Table 3.

**Table 3:** Percentage of hits in groups regarding items to evaluate children’s knowledge on breastfeeding according to pre- and post-intervention period. Recife, PE, Brazil, 2016

Items*	Pretest		P	Post-test (7th day)		p	Post-test (30th day)		p
	Control	Intervention		Control	Intervention		Control	Intervention	
	n (%)	n (%)		n (%)	n (%)		n (%)	n (%)	
1^†^	38(74.5)	40(83.3)	0.283^‡^	44(86.3)	44(91.7)	0.394^‡^	45(88.2)	45(93.8)	0.489^§^
2^||^	49(96.1)	46(95.8)	1.000^§^	49(96.1)	48(100)	0.495^§^	50(98)	48(100)	1.000^§^
3^¶^	37(72.5)	21(43.8)	0.004^‡^	41(80.4)	44(91.7)	0,108^‡^	38(74.5)	44(91.7)	0.024^‡^
4**	36(70.6)	31(64.6)	0.523^‡^	40(78.4)	38(79.2)	0,929^‡^	38(74.5)	44(91.7)	0.024^‡^
5^††^	46(90.2)	43(89.6)	1.000^§^	49(96.1)	46(95.8)	1.000^§^	49(96.1)	47(97.9)	1.000^§^
6^‡‡^	24(47.1)	31(64.6)	0.079^‡^	34(66.7)	42(87.5)	0.014^‡^	38(74.5)	46(95.8)	0.003^‡^
7^§§^	25(49)	37(77.1)	0.004^‡^	27(52.9)	47(97.9)	0.000^‡^	37(72.5)	47(97.9)	0.000^‡^
8^||||^	26(51)	21(43.8)	0.472^‡^	36(70.6)	40(80.3)	0.133^‡^	36(70.6)	40(83.3)	0.133^‡^
9^¶¶^	43(84.3)	39(81.3)	0.686^‡^	45(88.2)	46(95.8)	0.270^§^	45(88.2)	45(93.8)	0.489^§^
10***	42(82.4)	35(72.9)	0.259^‡^	44(86.3)	48(100)	0.013^§^	45(88.2)	47(97.9)	0.113^§^
11^†††^	26(51)	26(54.2)	0.751^‡^	28(54.9)	43(89.6)	0.000^‡^	27(52.9)	42(87.5)	0.000^‡^
12^‡‡‡^	18(35.3)	18(37.5)	0.820^‡^	30(58.8)	42(87.5)	0.001^‡^	33(64.7)	46(95.8)	0.000^‡^
13^§§§^	40(78.4)	36(75)	0.686^‡^	44(86.3)	43(89.6)	0.614^‡^	41(80.4)	46(95.8)	0.019^‡^
14^||||||^	47(92.2)	38(79.2)	0.064^‡^	49(96.1)	45(93.8)	0.672^§^	46(90.2)	48(100)	0.057^§^
15^¶¶¶^	49(96.1)	44(91.7)	0.358^‡^	50(98)	48(100)	1.000^§^	50(98)	48(100)	1.000^§^
16****	48(94.1)	48(100)	0.243^§^	49(96.1)	46(96.8)	1.000^§^	51(100)	48(100)	−^††††^
17^‡‡‡‡^	45(88.2)	39(81.3)	0.333^‡^	47(92.2)	46(95.8)	0.679^§^	48(94.1)	48(100)	0.243^§^
18^§§§§^	46(90.2)	44(91.7)	1.000^§^	45(88.2)	47(97.9)	0.113^§^	44(86.3)	44(91.7)	0.394^‡^
19^||||||||^	46(90.2)	43(89.6)	1.000^§^	48(94.1)	47(97.9)	0.618^§^	48(94.1)	48(100)	0.243^§^
20^¶¶¶¶^	45(88.2)	47(97.9)	0.113^§^	48(94.1)	48(100)	0.243^§^	46(90.2)	47(97.9)	0.206^§^
21*****	45(88.2)	37(77.1)	0.141^‡^	49(96.1)	47(97.9)	1.000^§^	48(94.1)	48(100)	0.243^§^

*Items: †1. The baby should be placed on the mother’s chest in the first hour after birth; ‡Pearson’s Chi-square test; §Fisher’s exact test; ||2. Breast milk makes the baby grows strong and healthy; ¶3. Breastfeeding is good for the mother’s health because it protects her against diseases; **4. Breastfeeding helps women’s body to recover faster after childbirth; ††5. Breastfeeding increases the affection between mother and baby; ‡‡6. Breast milk is always ready for the baby and it is free, unlike the milk sold in boxes or cans in the market; §§7. Breastfeeding protects the environment because it reduces the use of pacifiers, baby bottles, and milk boxes/cans that would be thrown into the trash; ||||8. Breast milk is a complete feed, and up to six months of life, babies should breastfeed only in the breast, they do not need to drink water, tea, juice, or eat porridge; ¶¶9. Babies who are fed with breast milk only have no schedule to breastfeed. They need to breastfeed several times a day/night; ***10. Breast milk is the only food your baby needs in the first six months of life; †††11. The use of the pacifier should be avoided, because it can interfere with breastfeeding; ‡‡‡12. The use of baby bottle can interfere with breastfeeding, and thus it should not be given to the baby; §§§13. Mothers can breastfeed a baby anywhere: at home and in public places such as squares; ||||||14. It is important for the father to be happy near his wife while breastfeeding; ¶¶¶15. Fathers can help the breastfeeding woman to do house chores such as sweeping the house; ****16. It is nice when the grandparents are happy with breastfeeding and help to take care of other grandchildren; ††††no statistical test was applied because there was 100% hits in both groups; ‡‡‡‡17. Grandparents can help the breastfeeding woman explaining how to breastfeed the baby; §§§§18. The son/daughter may help the breastfeeding mother by saying that breast milk is the best food for the baby’s health; ||||||||19. The family can help breastfeeding women by being happy with breastfeeding; ¶¶¶¶20. The nurse can help women explaining how to breastfeed the baby and clarifying doubts about breastfeeding; *****21. After six months the baby can continue to breastfeed and should start drinking juices and eating other foods.

In the interview on the 7th day, all children of the IG claimed to have played with the board game. Of these, 60.4% played less than six times, and 39.6%, six times or more. Children mentioned one or more people who played with them, 60.4% accounting for sister/brother; 41.7%, friends; 31.3%, mom; 14.6%, aunts; and 10.4%, fathers.

According to the score means regarding knowledge of the groups, considering the variables sex, age, exposure to breastfeeding, and number of times children played with the game, we found that: when it comes to sex, there was statistically significant difference when comparing the means among groups in the follow-up, and IG scholars had higher score mean for both sexes; on the 30th day, for the CG, girls had higher mean when compared with boys, with statistically significant difference; as for the age, there was statistically significant difference between the score means when comparing the groups, and higher means were found for IG schoolchildren, at the age group from 8 to 9 years, on the 7th and 30th days.

Exposing children to breastfeeding, verified at baseline, indicated statistically significant difference at follow-up, showing higher means concerning the knowledge score for the IG both on the 7th and on the 30th days. Regarding the children’s experience with the game, for the IG, we found statistically significant difference when comparing the score means on the 7th and 30th days; the higher score means regarding children’s knowledge were verified on the 30th day and comprised children who played with the game six times or more, according to Table 4.

**Table 4:** Mean values and standard deviation regarding the score of breastfeeding knowledge according to the variables sex, age, exposure to breastfeeding, and frequency with which children played with the game. Recife, PE, Brazil, 2016

Variables	Pretest		p-value	Post-test (7th day)		p-value	Post-test (30th day)		p-value
	Control	Intervention		Control	Intervention		Control	Intervention	
Sex									
Female	16.71±2.9	16.80±3.2	0.930*	17.86±2.7	20.20±1.2	0.001^†^	18.81±2.3	20.05±1.5	0.026^†^
Male	15.63±2.1	15.25±2.8	0.563*	17.40±2.5	19.32±2.0	0.003^†^	16.93±2.9	20.25±1.1	0.000^†^
p-value	0.134*	0.086*		0.440^†^	0.50^†^		0.016^†^	0.944^†^	
Age (years)									
8	16.36±2.1	15.82±3.5	0.612*	17.93±1.8	19.86±1.2	0.003^†^	17.86±2.1	20.45±0.9	0.000^†^
9	15.58±2.8	16.10±2.4	0.507*	17.23±3.0	19.71±2.0	0.001^†^	17.50±3.1	19.95±1.5	0.002^†^
10	16.91±2.3	15.40±3.8	0.340*	18.00±2.4	18.80±2.7	0.603^†^	18.00±2.9	19.80±1.3	0.223^†^
p-value	0.310^‡^	0.895^‡^		0.777^§^	0.804^§^		0.908^§^	0.364^§^	
Children’s exposure to breastfeeding									
Yes	15.87±2.5	16.00±3.0	0.818*	17.40±2.6	19.79±1.7	0.000^†^	17.56±2.8	20.19±1.3	0.000^†^
No	17.67±2.6	11.00	0.062*	19.00±1.4	15.00	0.130^†^	18.83±2.4	19.00	0.799^†^
p-value	0.102*	0.109*		0.161^†^	0.087^†^		0.268^†^	0.195^†^	
Frequency with which children played with the game									
<6	−	−	−	−	19.55±2.0	−	−	19.93±1.2	0.323^║^
≥6	-	-	-	-	19.89±1.3	-	-	20.53±1.2	0.039^║^
p-value	-	-	-	-	0.807^†^	-	-	0.027^†^	

*Student’s t-test; †Mann-Whitney test; ^‡^Analysis of variance; ^§^Kruskal-Wallis test; ^║^Wilcoxon test.

## Discussion

Our results confirm the hypothesis that children who participated in the educational intervention with the board game (IG) have higher score means concerning the knowledge on breastfeeding when compared with those who did not participate (CG). These findings corroborate the results of studies whose authors evaluated educational interventions aimed at children^7^ ^-^ ^8^ and teenagers^6^ ^-^ ^7^ ^,^ ^19^ ^-^ ^20^ attending school on the subject of breastfeeding, which reported increased knowledge for the treatment group.

For children of the CG, there was little increase in the score means of knowledge on breastfeeding, although they did not participate in the intervention. When comparing both groups, however, we found that for children of the IG, there was an increase in the score means higher than that for children of the CG.

Despite benefits of complementary breastfeeding and exclusive breastfeeding^1^, the early and inadequate introduction of liquids and other foods before the sixth month of the child’s life is common^21^. Types of foods vary according to age. In the first month, the supply of tea, water, juice, and non-breast milk prevails; in the sixth month there is increased consumption of all these foods, including fruits, porridge, and salty foods^22^.

According to schoolchildren aging from 5 to 11 years, in England, the infant feeding of babies has a variety of foods that may be offered individually or in combination. Formula feeding and baby bottle were the most mentioned when compared with breast milk Foods, such as porridge or purées made of fruits, vegetables, meats, and chocolate, have also been described^23^. Although these results are from the European continent, which has distinct characteristics when compared with Brazil, research conducted in the Brazilian scenario reported the inclusion of foods and other liquids in the baby’s feeding before the age of six months of life^21^ ^-^ ^22^. This indicates that this practice is common even in countries from different continents.

The inadequate children’s perception about infant feeding, with information contrary to breastfeeding^1^, may arise from the lack of knowledge about them and/or about practices observed in the contact with relatives in their daily lives. Concerning item 10, there was no statistically significant difference between the groups on the 7th day, and all the children of the IG marked the correct alternative, showing that it is possible to change inappropriate concepts with educational activities.

Another factor affecting the practice of exclusive or complementary breastfeeding is the use of artificial nipples (pacifier and baby bottle). Pacifier is a risk factor for early interruption of exclusive breastfeeding, and the use of artificial nipples is associated with the lack of breastfeeding after six months of the child’s life^24^ ^-^ ^25^.

More than half of children surveyed at the pretest concerning the use of artificial nipples, in both groups, said that pacifiers should be avoided in such a way not to harm breastfeeding (item 11). However, the percentage of hits regarding the use of baby bottle (item 12) was low, accounting for about 35% at baseline. After intervention, there was a significant increase in the correct answers of both items for the IG, which accounted for a percentage of hits close to or above 90%. These results, referring to the pretest, may indicate that children have contact with information on breastfeeding by the media, the family, and the society. These children may have learned that pacifiers should not be used; on the other hand, they must have noticed or even used a baby bottle as a means to feed themselves^9^.

Another noteworthy aspect is item 13, concerning breastfeeding in public, which obtained high percentage of hits at baseline in both groups. However, on the 30th day, we identified statistically significant difference, since there was an increase in the number of children for the IG who responded correctly.

The perception of breastfeeding in public as embarrassing or less acceptable when compared with breastfeeding in a private environment or from people close to the mother is mentioned by children^9^, teenagers^19^ ^-^ ^20^, and adult men^26^ in Brazil, London, and in the United States of America. Such evidence indicates that in several cultures breastfeeding in public can cause discomfort to people. Therefore, it is important to discuss this practice since childhood in order to attribute new meanings to it, as a practice that is natural and physiological in such a way it becomes natural later in the adulthood.

The support of the family in the breastfeeding process was also a topic discussed with female teenagers at a high school in Taiwan during an educational intervention in the classroom. Such activity promoted a significant increase of score means concerning breastfeeding-related knowledge and attitudes in the experimental group until a month after the intervention^6^.

Items 14 to 21 broached the support of members of the women’s primary (partner/father, grandfather, grandmother, child) and secondary (nurse) networks, which accounted for high percentages of hits at baseline, ranging from 77.1% to 100% in both groups. This indicates that children perceive the support actions - emotional, instrumental, in person, informative, and self-help^4^ ^)^ - required for women during breastfeeding and which are sensitive to help them if properly instructed by others, from the care aimed at the baby to the sharing of knowledge acquired at school.

Members of the primary social network often advise women on the infant feeding of the child, usually based on their beliefs, attitudes, and previous experiences with such practice^27^. In this network, they can provide support by assisting women in the household chores and child care. When observing difficulties or need for more information on the management of breastfeeding, women can be advised to seek help from health professionals of the secondary network^28^.

The school is the ideal place to discuss breastfeeding and to demonstrate to young people the importance of such behavior to health^6^, helping to demystify myths and beliefs unfavorable to breastfeeding and to resume it as something natural and physiological. This may reflect on the education of adults more apt to support this practice in the future^7^, especially concerning the positive and active support of the baby’s father or partner who influences on the self-confidence of women in breastfeeding^29^.

Several studies^7^ ^-^ ^9^ carried out with children and adolescents from elementary and high schools have shown positive effect of interventions on knowledge, attitudes, behavior, social norms, support to women during breastfeeding, and intention in breastfeeding in the future. These interventions were diverse and included one or more sessions of discussion at the classroom on breastfeeding by lectures using slides, videos, role-playing, and interactive games^7^ ^-^ ^9^.

Indeed, health education interventions involving activities can improve children’s knowledge on healthy lifestyle habits, which is essential to motivate behavioral changes. However, in addition to playful interventions, other strategies must be implemented for the effective behavioral change to occur^30^.

In our research, we identified a significant increase, in the post-test, in the means of children’s knowledge for the IG within the age group between 8 and 9 years, both on the 7th and on the 30th days; and concerning the girls, for the CG, on the 30th day. Thus, the board game strategy, for the IG, was more suited to younger children. Furthermore, breastfeeding is closer to the everyday reality of girls^8^, which may have influenced, somehow, on the increase in the knowledge of these students for the CG.

Moreover, the age group between 7 and 10 years covers the period of concrete operations in which children have greater understanding of logical relations and increased interest in collective games^13^ ^-^ ^14^. Hence, in our research, youngest schoolchildren were possibly more receptive to the board game due to their interest in this type of technology.

Considering the use of an educational technology, a board game, and the fact that the children have taken the toy at home enabled additional sessions of the game with their family and friends, which may have favored knowledge acquisition, considering children who played six times or more reached highest score means on the 30th day.

The educational game intended for the family turns out to be a nice resource able to promote the discussion of topics important to the health of children and adolescents, and the active learning of contents that may, perhaps, reflect in the adoption of healthier behaviors in adulthood^31^ ^-^ ^32^. Therefore, our results can be attributed to the type of intervention - board game -, which allowed teaching about breastfeeding in a playful and enjoyable way, with the active participation of children, involving interaction with their family and friends, thus favoring knowledge acquisition by enabling exchanges of experiences and learnings.

According to the theory of cognitive development, learning comprises children’s experience with the object and the environment, in which there is a process of imbalance and recovery of balance from adaptation, assimilation, and accommodation. The use of game-based educational tools favors the children’s learning because it assists in the creation of schemes and in the apprehension of knowledge in the memory^33^. As new contents were presented throughout the board game, the balance process was restarted, until the cognitive development occurred, resulting in the accommodation of knowledge on breastfeeding, which was verified by increased score means for the IG.

Social interaction and mediation are also essential components in the didactic-pedagogic process for the assimilation of knowledge to occur. Participation of the teacher, an adult, parents, and their own colleagues in the pedagogical activity allows children to establish cooperative relationships with people who have different knowledge according to their age, experiences, and level of cognitive development. This social interaction encourages the children’s learning, favoring advances in the knowledge assimilation that would not spontaneously occur in an isolated way^14^.

In our study, the mediation of a person who applied the intervention, the social interaction provided by the board game, as well as the repetition of the game at home with other family members and/or people from children’s social network, enabled the establishment of schemes and the accommodation of the content concerning breastfeeding.

However, other factors were also important for this process, because, from the technology design step to the application of the intervention, we were careful with several aspects related to the game, contents, and the technology approach, aiming to achieve the cognitive development^33^.

In this context, a board game addressing oral health, when compared with a didactic activity using cards between schoolchildren aged between 5 and 10 years, proved to be more effective in increasing students’ knowledge in younger age groups (5 to 7 years)^12^. It is possible that the search for pleasure and entertainment have stimulated children to play more often with the board game, providing social interaction with other people and fostering the cognitive development^13^ ^-^ ^14^.

Regarding children’s exposure to breastfeeding, we observed that most children in both groups claimed to have been breastfed and have witnessed a woman breastfeeding. Children are able to respond if they were breastfed or if they saw this practice in their social environment^8^, to describe and to draw scenes in which women breastfeed at home^23^. Therefore, they are exposed to breastfeeding by being aware they were breastfed as a baby and identifying this practice in their family everyday lives or in their social environment, which may corroborate the choice for breastfeeding in their adulthood lives.

## Conclusion

The board game as educational technology was effective in increasing children’s scores of knowledge on breastfeeding for the IG, which we verified on the 7th day post-intervention and continued until the 30th day.

Our results are limited to the follow-up period and to the children’s learning about breastfeeding-related contents and, therefore, do not extend to behavioral changes.

Hence, we suggest new analytical studies to evaluate the long-term effect of educational interventions on children’s knowledge on breastfeeding, on the support provided to women regarding the breastfeeding practice, in order to compare other teaching methods and qualitative studies to thoroughly investigate the breastfeeding phenomenon according to children’s point of view.
